# Supplementary material for: The effect of motivational and instructional self-talk on attentional control under noise distraction
Source: PLoS One. 2023 Sep 29;18(9):e0292321. doi: 10.1371/journal.pone.0292321 (PMC10540956; doi:10.1371/journal.pone.0292321)
Supplement: Attachment — Submitted filename: Response to Reviewers.docx [file pone.0292321.s003.docx]

**Response to reviewers**

Dear Editor and Reviewers,

We would like to thank the reviewers for carefully reading our manuscript. We appreciate the comments and suggestions. In the following, we include a point-by-point response to the comments from each reviewer. In the revised manuscript, all the changes have been highlighted in red.

Reviewer #1: Comments to the Author:

General comments:

This study explored the effects of noise distraction on attentional control in basketball players and the moderating effects of self-talk. I think this study has practical implications. Firstly, the attentional control theory was verified in a specific sport (basketball). Secondly, the results were validated using eye-movement equipment. Finally, no positive results were found is insightful, but the authors do not discuss it in depth.

Specific comments:

Authors need to enhance the writing norms:

1. Line numbers should be added throughout.

**Thank you for your suggestion. However, I did submit a manuscript with the line numbers, maybe the version you reviewed doesn't have the line numbers.**

2. Space before and after the symbols ±, =, etc.

**Thank you for your suggestion. I have added the space around all symbols.**

3. English writing should be left-aligned, not end-to-end.

**Thank you for your suggestion. I have changed the text into left-aligned.**

4. Pay attention to the tenses.

**Thank you for your suggestion. I have reviewed the tenses paragraph by paragraph and marked the changes in red.**

5. Note the subject of a sentence.

**Thank you for your suggestion. I have changed the grammar errors and marked the changes in red.**

6. Please distinguish between full and half corner punctuation, e.g., for the Pupil diameter section of P16, please use a half corner comma.

**Thank you for your suggestion. I have changed the mis-usings and marked the changes in red.**

About abstract:

The abstract should include purpose, methods, results, and conclusions.

1. There is too much "purpose". Consider stating that basketball players' attentional control affects performance, especially in the presence of noise distractions. Improving attentional control is therefore necessary. Self-talk is a psychological skill that enhances motor and cognitive performance and may improve attentional control. Thus, our study investigated the effects of noise distractions on attentional control and the moderating effect of self-talk.

**Thank you for your suggestion. I have removed redundant parts to decrease the repetition of the "purpose" and added a sentence that illustrates how noise from competitive conditions can affect the attentional control (AC) and performance of basketball players. Please see the revised abstract for details.**

2. Missing "Method", consider adding: (a) How does noise distractions operate? (b) What did the Stroop task and the Antisaccade task measure respectively? (c) Were instrumental and motivational self-talk used in each experiment?

**Thank you for your suggestion. (a) In the abstract, I have mentioned that participants were required to wear earphones to receive noise distraction; (b) Both the Stroop and Antisaccade task are means of measuring AC, as I mentioned in the last paragraph of “Introduction”(p.8): “AC will be assessed using the Stroop task and Antisaccade task, two well-established paradigms for investigating AC.” Hence, I added “In Study 1, the effects of noise distraction on AC were initially demonstrated” in abstract; (c) the self-talk interventions only used in study2. I think “In Study 1, the effects of noise distraction on AC were initially demonstrated” this sentence can not only tell readers what the task was measuring, but also tell them that self-talk was not used in Study1.**

3. The "conclusions" are not sufficiently illuminating. The current conclusions merely repeat the results. And your study did not find these findings.

**Thank you for your suggestion. I have rewritten the conclusion according the results: “In conclusion, noise distraction increases cognitive load and reduces AC. For basketball players, the implementation of instructional self-talk in quiet condition proved to be more effective in enhancing AC. However, the utilization of motivational self-talk in noise distraction condition diminished AC level.”**

About introduction:

1. The first paragraph is too long. Consider stopping after the introduction of attentional control theory and discussing the effect of noise on attentional control in the next paragraph.

**Thank you for your suggestion. I have started a new paragraph to discuss effect of noise on AC.**

2. One of the innovative points of this study is to examine the effect of noise distractions on attentional control and the moderating effect of self-talk among basketball players. Therefore, there is a need to emphasise the specificities of basketball, such as where does basketball need to focus attention? What distractions need to be inhibited more than anything else?

**Thank you for your suggestion. I have added three major information in p.5, the first one is illustrating two dimensions in sport; the second discusses what needs to be focused on in basketball, and the third explains what distractions need to be avoided in basketball. Please refer to the revised introduction for more details.**

3. d values in italics, spaces before and after the “ = ”; what is the exact value of the 95% CI for the meta-analysis of Szalma et al. (2011)?

**Thank you for your suggestion. I have changed “d” in italics and added space around “ = ”, plus, I have added the exact value of the 95% CI . Detail in “mean effect size across the 191 cognitive tasks was *d* = -0.34 (95%CI = -0.42 to -0.25)** **with the highest effect size for high loudness, short duration, *d* = -0.68, (95%CI = -0.96 to -0.40).”**

4. The paragraph introducing the quiet eye is oddly placed, splitting the review of the effects of noise distractions on attentional control. It could be considered for placement after the literature (Derakshan et al., 2009).

**Thank you for your suggestion. I have moved the paragraph which introduces the relation between quiet eye and performance after the first paragraph. Surprisingly, it seems to have made the logical chain more fluent. Thank you for this suggestion again.**

5. Note that the key words noise interference, noise disturbance, noise distraction should not be substituted at random.

**Thank you for your suggestion. I have replaced all “noise interference” and “noise disturbance” with “noise distraction”.**

6. Regarding the mechanisms by which self-talk affects motor performance, at citation points [22-26], it should be emphasized or summarized that the mechanisms are improvements in attention rather than listed theories and research findings.

**Thank you for your suggestion. I have summarized** **the mechanisms by which self-talk affects motor performance through attention and added this sentence after introducing the research findings: “In summary, it can be concluded that self-talk affects performance by AC, and the correct use of self-talk facilitates the dominance of the goal-directed attention system, leading to AC on the task at hand.”**

7. "Hase et al. (2019) found motivated self-talk to be better due to participants' greater willingness to perceive threat as a challenge." What is the illumination of this finding for your research? One might consider adding: the study suggests that whether participants perceive the task as a threat or a challenge influences the performance of self-talk types on fine motor control.

**Thank you for your suggestion. This sentence is helpful to better illustrate the illumination of this finding for my research, and I have added it (details in p.7).**

8. The mechanism by which self-talk improves basketball performance needs to be highlighted. And it needs to be stressed that attentional control is key.

**Thank you for your suggestion. At the beginning of our discussion, I hope we could build a consensus is that “key” means the most important thing. As I responded in suggestion 6, I have added description that self-talk affects performance by attention. However, there are lots of aspects that self-talk affects performance. I acknowledge that AC is the key in the present research, but other factors (e.g., motivation, motor execution) are equally important to basketball performance. Therefore, I highlighted the mechanisms by which self-talk affects motor performance through attention, but stressing that AC is the “key” of performance may not be necessary.**

9. Please explain why two paradigms are used for study 1 and study 2?

**Thank you for your question. The reason why used two paradigms separately is avoiding common method biases. If I used the same task to measure AC in two studies could potentially lead to common method biases. This may lead to similar or correlated measurement errors that are specific to the task.** **Using different tasks to measure AC in two studies can help reduce the impact of common method biases.**

10. The introduction needs to explain what can be examined in study 1 and study 2 respectively.

**Thank you for your suggestion. Study1 and Study2 both examined AC level and I mentioned it in the last paragraph of Introduction: “AC will be assessed using the Stroop task and Antisaccade task, two well-established paradigms for investigating AC [8, 31, 32]. Study 1 will measure the effect of noise distraction on AC via the Stroop task, and the Study 2 will further examine the effects of noise distraction on basketball players and the moderating effect of self-talk via the Antisaccade task.”**

11. Is “Participant” a normal university student?

**Thank you for your question. The “participants” in hypothesis refer to all participants in Study 1 and Study 2. The participants in Study 1 are normal university students, and participants in Study 2 are basketball players, and I hypothesized that their AC will be affect by noise distraction, so I call them “participants” collectively.**

12. Hypotheses 2 and 3 should count as one hypothesis: self-talk moderates the effect of noise distractions on attentional control. Specifically, with noise distractions, only instrumental self-talk group improved attentional control compared to the other groups.

**Thank you for your suggestion. Hypotheses 2 and 3 are indeed redundant expressions, I have changed the original hypothesis as your suggestion. Please see the last paragraph of introduction for more details.**

13. The use of different participants and different paradigms made the logic of the two experiments incoherent. And as previous studies have demonstrated that noise can affect attentional control, study 1 alone can be considered as a basis for testing the validity of the material.

**Thank you for your suggestion. As I responded in Suggestion 9 and 10, the Stroop and Antisaccade task are well established paradigms for testing AC, and the purpose of using different paradigms is avoiding the common method biases. Therefore, using different paradigms may not made the logic of the two experiments incoherent. However, you are totally correct about considering Study 1 as a basis for testing the validity of the material, and this is the most important reason why I designed it.**

Regarding study 1:

1. Are the participants normal university students? Need to specify.

**Thank you for your question. Yes, they are normal university students. I have specified this in the part of participants.**

2. Lowercase z in Hz.

**Thank you for your suggestion. I have changed the mis-usings.**

3. Clarification is needed on how the results of the Stroop task can be interpreted. For example, in the inconsistent condition, shorter response times indicate stronger inhibition and smaller error rates indicate stronger inhibition.

**Thank you for your suggestion. I have added the interpretation of indicators in p.10.**

4. Is the validity of noise material tested?

**Thank you for your question. As I responded in Suggestion 13, the purpose of Study 1 is testing the validity of noise material. The error rate was higher under noise distraction condition, it indicated that the material is effective.**

5. Instead of “greater”, use “more” to describe the size of a number in Data analysis.

**Thank you for your suggestion. I have replaced “greater” with “more”.**

6. Why use data within 2 standard deviations instead of 3?

**Thank you for your suggestion. There are two reasons why I consider 2 and -2 as thresholds. First, a z-score threshold of 2 is more commonly used than 3 generally, because using a z-score threshold of 3 would result in a more conservative criterion for identifying outliers, as it would require data points to be more extreme than with a z-score threshold of 2. Using data between 2 and -2 z-score can increase the reliability of statistical analyses by reducing the influence of extreme values on the results. Second, there may be situations where a z-score threshold of 3 is appropriate, such as when the data are highly skewed. However, the distribution of data is normal, so the use of 3 as threshold is not considered.**

7. For images and tables please use the insert form [insert figure 1], then the images and tables at the end of the manuscript.

**Thank you for your suggestion. However, I insert images and tables follow the “PLOS Manuscript Body Formatting Guidelines”.**

8. Please use the 3-wire table

**Thank you for your suggestion. However, the formal of tables follows “PLOS Manuscript Body Formatting Guidelines”.**

Regarding study 2:

1. Why did study 1 and study 2 use two different paradigms?

**Thank you for your question. I have answered this question in suggestion 9 and 13.**

2. Is the qualified rate equal to the correct rate?

**Thank you for your question. Yes, the qualified rate is equal to the correct rate.**

3. Were the sample sizes of study 1 and study 2 tested a priori power by G*power?

**Thank you for your question. The sample sizes were calculated before the experiments, and the relevant information has been added to the "participants" section. Specifically, 34 participants were included in Study 1 and 36 participants were included in Study 2, and all sample sizes were met.**

4. In Procedure (Page 13), it should be “study 1” and not “experiment 1”.

**Thank you for your question. I have changed this mistake and marked it as red.**

5. Stress assessment is a variable that the researcher should control for.

**Thank you for your feedback and suggestion. While we did not control for stress in this study, we acknowledge that stress can be an important factor to consider in present research. In hindsight, we agree that controlling for stress could have strengthened the internal validity of our study. Therefore, the first limitation we proposed in discussion is lacking measurements of control variables.**

**In future research, we plan to control for stress by implementing a stress induction procedure or by measuring participants' stress levels prior to the experiment. We will also report any stress-related variables in our study to better understand how stress may have influenced our results. Thank you for your valuable feedback and I will make sure to address this issue in future research.**

6. “The rest of group” can be replaced with “other groups” in the P15 manipulation check.

**Thank you for your suggestion. I have replaced “the rest of groups” with “other groups” in p.17.**

7. Note the writing specification, (F(1, 36) = 2.20, p =.147, ηp2 = .058), these data can be enclosed in parentheses.

**Thank you for your suggestion. I agree that enclosing these data in parentheses would improve the clarity and readability of the manuscript. I will make the necessary changes accordingly in the revised version of the manuscript.**

8. Antisaccade error rate section, the reader is more interested in knowing the difference between motivational and instrumental self-talk in the presence of noise distractions. Rather than just reporting significant results, non-significant results may sometimes be more meaningful.

**Thank you for your suggestion.** **I agree that this is an important point to clarify for the reader and I believe that it is still important to report this finding as it provides important information for future research in this area. I will revise this section to include this information. Please see more details in p.19.**

9. Has consideration been given to setting some additional variables as covariates to control for the interference? For example, the Stressor Appraisal Scale (Schneider, 2008) was used to measure whether participants perceived the task as stressful or challenging.

**Thank you for the suggestion to set additional variables as covariates to control for interference. However, due to limitations in our study design, I am unable to collect data on those variables. As a result, I am not able to include them as covariates in our analysis. I acknowledge that this may be a limitation of our study and will address it in future research.**

10. Please italicise statistics, e.g., M, SD.

**Thank you for your suggestion. I agree that italicizing statistics such as M and SD will enhance the clarity and readability of the manuscript, and I have made the necessary changes accordingly.**

11. There are no table notes in Table 2. The abbreviation of each table must be indicated so that its meaning is clear when it appears independently.

**Thank you for your comment regarding Table 2. I appreciate your feedback and recognize the importance of indicating the meaning of abbreviations used in the table. I have added table notes to clarify the meaning of abbreviations and improve the readability of the table.**

12. Please standardise the font for captions and notes on tables and images.

**Thank you for your suggestion. I have revised our manuscript to ensure that all captions and notes have the same font size and type.**

13. The relationship between quiet eye and eye movement data needs to be explained.

**Thank you for your question. Although quiet eye and eye movements in the antisaccade task are both indicators measure AC, thy are two different concepts related to eye behavior.**

**Quiet eye refers to the final fixation of the eyes on a specific target before initiating a motor action, such as throwing a ball or shooting a target in sports. During the quiet eye phase, the athlete's gaze is fixed on a specific location, and this gaze fixation is associated with improved performance accuracy.**

**On the other hand, eye movements in the antisaccade task refer to a cognitive task that requires inhibiting a reflexive saccade toward a suddenly appearing visual stimulus and instead making a voluntary eye movement to the opposite side. The task requires inhibitory control and cognitive flexibility, and performance on this task is used as an index of executive function.**

**Therefore, the key difference between quiet eye and eye movements in the antisaccade task is that the former refers to a gaze fixation behavior associated with improved motor performance accuracy, while the latter refers to a cognitive task requiring inhibitory control and cognitive flexibility.**

About discussion:

1. “However only to a limited extent were hypotheses 2 and 3 supported.” is not quite the appropriate sentence, because it is not supported.

**Thank you for your suggestion. I have changed another expression based on new hypothesis 2 that according to Suggestion 12. Please see more details in p.20.**

2. “Specifically, while there was a significant main effect of self-talk on antisaccade error rate, post-hoc comparisons revealed that antisaccade error rate of motivational self-talk group was significantly higher than control group (p =.028) and instructional self-talk group (p =.014).” should be in the results section, not the discussion section.

**Thank you for your suggestion. I agree that the information regarding the significant main effect of self-talk on antisaccade error rate and the post-hoc comparisons should be included in the Results section rather than the Discussion section. I have revised the manuscript accordingly to reflect this change. Please see more details in p.18 and p.21.**

3. These studies “Galanis et al. (2018), Gregersen et al. (2017), Galanis et al. (2021)” can only show that self-talk improves attentional control. You need to discern why your study did not find that self-talk improves attentional control. You need to clarify the differences between previous study and your study, which may be that the task is different in difficulty, or that certain confounding variables have not been controlled for.

**Thank you for your valuable feedback. I agree that it is important to clarify the differences between our study and previous studies that have found self-talk to improve attentional control. I have added two sentences in p.22 to address this reason. While it is difficult to directly compare the task difficulties between studies, our study was unique in that we distinguished between motivational and instructional self-talk interventions. This may have contributed to the partially different findings compared to previous studies. Thank you again for your helpful comments.**

4. The focus needs to be on “self-talk as an internal disturbance”.

**Thank you for your suggestion. I apologize for misapplying the interpretation of self-talk as an internal disturbance in the present research. It is important to note that self-talk can be either positive or negative, and its impact on an individual's mental state and performance can depend on the content and context of the self-talk. While some types of negative self-talk, such as self-criticism or rumination, can contribute to feelings of distress or inner disturbance, the types of self-talk used in the present research were motivational and instructional self-talk, which are typically viewed as positive forms of self-talk.**

**Regarding the reference I quoted, Brinthaupt (2019) suggested that although self-talk frequency is positively correlated with cognitive disruption, the use of self-talk is generally viewed as a self-regulatory tool. Additionally, Ariel (2022) found that some individuals may view self-talk as an inner disturbance due to the social stigma associated with it as non-normative behavior in adulthood. However, it is important to recognize that self-talk is a natural and important cognitive process that individuals use to regulate their thoughts and emotions (Ariel, 2022).**

**Therefore, while self-talk can sometimes be viewed as an internal disturbance, it is not a correct interpretation in the present research. I have deleted this expression from the manuscript, and I appreciate your feedback and guidance in improving the clarity of our interpretation.**

5. Is high motivation equal to motivational self-talk? I think high motivation is the outcome and motivational self-talk is the method. I do not think that the literature that exemplifies how high motivation impairs attentional control proves that motivated self-talk is bad. If you think my understanding is incorrect, please illustrate with examples of literature.

**Thank you for your question. You are correct that high motivation is an outcome of motivational self-talk, as motivational self-talk tends to increase motivation. In the present research, basketball players who used motivational self-talk performed worse in the Antisaccade task compared to other groups. One possible explanation for this is that the high motivation brought about by self-talk may have influenced attentional control (AC).**

**First,** **athletes generally have a higher level of motivation than the general population, which is one of their special characteristics (Giakoni-Ramírez et al., 2022; Šmela et al., 2017).**

**Second, according to overmotivation theory (also known as Incentive-based theory), people are motivated to engage in certain behaviors or activities because they believe that doing so will lead to a desirable outcome or reward. However, larger and more desirable rewards are more likely to motivate people than smaller or less desirable ones. Overmotivation from rewards can impair performance even in simple motor tasks,** **and can also lead to a narrow focus on achieving the desired outcome at the expense of other important factors, such as skill development and enjoyment of the activity (Mobbs et al., 2009; Short & Sorrentino, 1986).**

**Third, there is evidence to suggest that high approach-motivation reduces the breadth of attention (Gable & Harmon-Jones, 2008).** **Breadth of attention is an important factor in the Antisaccade task because it reflects an individual's ability to maintain attentional focus on the central fixation point while also monitoring the periphery of their visual field for the appearance of the target stimulus. This requires individuals to have a broad attentional focus and the ability to suppress distractions that may interfere with their task performance.**

**In conclusion, one possible reason that basketball players who used motivational self-talk performed worse in AC than other groups is the conjunction of high motivation in athletes and high motivation from self-talk interventions, which may have resulted in a decrease in AC.**

**Your question has helped me provide a better explanation in the discussion section regarding this phenomenon. Please see p.24 for more details.**

6. The strength of your study is the addition of eye-movement data to prove your research hypothesis, but this aspect of the explanation is rarely mentioned in the discussion and needs to be added.

**Thank you for your suggestion. The antisaccade task has some advantages compared to the Stroop task or traditional attentional control tasks. Firstly, the task requires a high degree of cognitive control, as it involves inhibiting a prepotent response (looking towards a peripheral target) and instead generating a saccade in the opposite direction. This makes it a more ecologically valid measure of attentional control than traditional tasks, as it more closely reflects the types of inhibitory processes required in real-world situations where we need to inhibit automatic responses. Second, the antisaccade task has been shown to be a reliable and valid measure of attentional control, with good test-retest reliability and sensitivity to individual differences in attentional control. the two core indicators of the antisaccade task are saccade latency and saccade error rate, which correspond to the reaction time and error rate of key presses in the Stroop task, other eye movement data (except pupil diameter) is meaningless to us. Pupil diameter is an additional indicator to measure cognitive load.**

**However, I completely agree that discussing pupil diameter data is an essential aspect of our research, which adds strength to our study and proves our research hypothesis. I have added related discussion, please see p.24 to p.25 for more details**

7. “Second, the present study provides guidance on the use of self-talk as a psychological skill for basketball players to improve AC”. Your study did not prove this, but only found that self-talk impairs attentional control. So, you should stress that self-talk can be a distraction and in practice you need to be aware that not all mental skills are applicable.

**Thank you for your suggestion. I have changed the expression into “Second, the present study …… to maintain AC under distraction conditions.” And in the final of Discussion, I proposed ND-ST-AC Integration Model to better illustrate the results. Please see more details in p.25 and Fig 4.**

8. I see that your limitations mentioned not satisfying G*power, but in the methods section also mention how much sample size should be used for the calculation using G*power. In addition, you can perform a post hoc power calculations to determine the power.

**Thank you for your suggestion. I have included the information related to the sample size calculated by G*power. Furthermore, after performing a post hoc power analysis, Power (1-β err prob) = 0.768. This indicates that the current study has a relatively high probability of detecting a true effect or relationship, provided that such an effect or relationship exists in the population and the statistical test is appropriately designed and executed.**

**References**

Ariel, N. (2022). Don’t think before you speak: on the gradual formation of thoughts during speech. *Pedagogy, Culture &amp; Society*, 1-13. https://doi.org/10.1080/14681366.2022.2039270

Brinthaupt, T. M. (2019). Individual Differences in Self-Talk Frequency: Social Isolation and Cognitive Disruption. *Frontiers in Psychology*, 10(1088). https://doi.org/10.3389/fpsyg.2019.01088

Gable, P. A., & Harmon-Jones, E. (2008). Approach-Motivated Positive Affect Reduces Breadth of Attention. *Psychological Science*, 19(5), 476-482. https://doi.org/10.1111/j.1467-9280.2008.02112.x

Giakoni-Ramírez, F., Merellano-Navarro, E., & Duclos-Bastías, D. (2022). Professional Esports Players: Motivation and Physical Activity Levels. *International Journal of Environmental Research and Public Health*, 19(4), 2256. https://doi.org/10.3390/ijerph19042256

Mobbs, D., Hassabis, D., Seymour, B., Marchant, J. L., Weiskopf, N., Dolan, R. J., & Frith, C. D. (2009). Choking on the Money: Reward-Based Performance Decrements Are Associated With Midbrain Activity. *Psychological Science*, 20(8), 955-962. https://doi.org/10.1111/j.1467-9280.2009.02399.x

Short, J.-A. C., & Sorrentino, R. M. (1986). Achievement, affiliation, and group incentives: A test of the overmotivation hypothesis. *Motivation and Emotion*, 10(2), 115-131. https://doi.org/10.1007/BF00992251

Šmela, P., Pačesová, P., Kraček, S., & Hájovský, D. (2017). Performance Motivation of Elite Athletes, Recreational Athletes and Non-Athletes. *Acta Facultatis Educationis Physicae Universitatis Comenianae*, 57(2), 125-133. https://doi.org/10.1515/afepuc-2017-0012

Reviewer #2: p.5. The authors are advised to add in the self-talk literature that there is empirical evidence that self-talk strategies are effective in enhancing sport/task performance through systematic (Tod et al., 2011) and meta-analytic (Hatzigeorgiadis et al., 2011) reviews. Also regarding the matching hypotheses the authors are advised to consider two more matching hypotheses; one involving the setting by self-talk type matching and one involving the learning stage by self-talk type matching. Regarding the former, motivational self-talk seems more appropriate in competitive settings, whereas instructional self-talk seems more appropriate in training settings(Hatzigeorgiadis, Galanis, et al., 2014). Regarding the latter, instructional self-talk should be more effective for novel tasks, or tasks at the early stages of learning, whereas motivational self-talk should be more effective for well-learned tasks, or tasks at the automatic stage of performance (Zourbanos, Hatzigeorgiadis, Bardas, & Theodorakis, 2013).

**Thank you for your suggestion. We appreciate your input and have revised our paper to include the empirical evidence of the effectiveness of self-talk strategies in enhancing sport/task performance through systematic and meta-analytic reviews (Tod et al., 2011; Hatzigeorgiadis et al., 2011).**

**While we agree with the two hypotheses you proposed, we would like to clarify that they may not be suitable for the present study due to two specific reasons. First, the condition settings in the present study are quiet and noise distraction conditions, which may not fully match with training and competitive settings. There may be external distractions in the training setting, while the quiet laboratory environment in our study meant that external distractions were well-controlled.**

**Second, in Study 2, during the practice stage, if the error rate of the first saccade was higher than 20%, a new 20-trial was required until the error rate was less than 20%. This means that all participants in Study 2 had well-learned the purpose of the antisaccade task, and therefore the hypotheses involving the learning stage may not match the present study design.**

**However, these two hypotheses you proposed were perfect to add to the introduction to better introduce motivational and instructional self-talk, thank you again for your insightful suggestion. Please see more details in p.6 to p.7.**

p.7. Although in the introduction the authors write also about motivational self-talk in the hypotheses motivational self-talk is not clearly reported.

**Thank you for your suggestion. We have revised the manuscript to better illustrate the hypotheses at the end of Introduction, please see more details in p.9.**

p.8 Why the authors didn’t use control group in the experimental design. Can this added in the limitation section?

**Thank you for your suggestion. We appreciate your attention to detail and concern regarding our experimental design. We would like to clarify that we did, in fact, use a control group in our study. Participants in the control group performed the same task as the experimental group but did not receive any self-talk intervention. We apologize for any confusion or lack of clarity in our paper that may have led to this misunderstanding. We will make sure to clarify this in the revised manuscript. Thank you for bringing this to our attention.**

p.10. Are the participants in experiment 2 participated also in experiment 1?

**Thank you for your question. The participants in Experiment 2 did not participate in Experiment 1, as we recruited a new set of participants for each experiment. The participants in Experiment 1 were normal university students, the participants in Experiment 2 were national second-level basketball players. I have clarified this in the revised manuscript**

p.11. Have the authors tested the assumptions of the mixed design?

**Thank you for your question. A Mauchly's sphericity test was conducted to examine the assumption of sphericity for the within-subjects factor in a mixed design. The result showed that for all variables, the Mauchly's W value was 1.000, indicating that the assumption of sphericity was met. The approximate chi-square value was .000 with 0 degrees of freedom, but the significance level could not be calculated. Therefore, no adjustment was necessary and the traditional F-test could be used to analyze the data.**

**In conclusion, the assumption of sphericity for the within-subjects factor was met based on the result of the Mauchly's sphericity test.**

p.13. Why did you use these self-talk cues? How did you select them? The authors are advised to give more details about the self-talk cues selection.

**Thank you for your question. Thank you for your suggestion. We selected the self-talk cues based on the working definition of self-talk (Hardy, 2006) and previous literature (Abdoli et al., 2018; Dali & A. Parnabas, 2018). According to one of definitions from Hardy (2006), self-talk should serve at least two functions: instructional and motivational, for the athlete. Therefore, instructional self-talk cues and motivational self-talk cues were used in the present research.**

**“I can do it” or “I can” is a** **commonly used motivational self-talk cue that has been shown to enhance performance among basketball players (Abdoli et al., 2018; Dali & A. Parnabas, 2018).**

**There are no references about the instructional self-talk cue on the antisaccade task. However, according to Hardy (2006), the performance of relatively fine motor tasks requiring skill, timing, and accuracy was enhanced to a greater extent by instructional self-talk, focusing on the technical aspects of performance, than motivational self-talk. Therefore, “Look at the opposite” is intended to remind participants to look in the opposite direction of the target, which is the goal and technical aspect of the task.**

**We have added these details to the revised manuscript to provide more clarity on the selection of the self-talk cues.**

p.13. Regarding the manipulation check, did you ask the participants if they said

**Thank you for your question. We did ask participants after the experiment whether they used any self-talk cues other than the ones provided to them (i.e., "I can do it" for the motivational self-talk group and "look at the opposite" for the instructional self-talk group). None of the participants reported using any other self-talk cues during the task. We have added this information to the manuscript in p.17**.

p. 14. Have the authors tested the assumptions of 2x3 repeated measures anova?

**Thank you for your question. Like I responded in “p.11. Have the authors tested the assumptions of the mixed design?”,** **the assumption of sphericity for the within-subjects factor was met based on the result of the Mauchly's sphericity test.**

p.15. Please report the results of the assumptions of 2x3 Manova?

**Thank you for your suggestion. Here is the report the results of the assumptions of 2x3 MANOVA.**

**Antisaccade Latency: The 2 × 3 RMANOVA showed that neither Condition (F (1, 36) = 0.206, p = .651, η2 p = .069) nor Group (F (2, 36) = 2.651, p = .077, η2 p = .073) had a main effect on antisaccade latency. Furthermore, there was not a significant Condition × Group interaction (F (2, 36) = 0.066, p = .936, η2 p = .002) either**

**Antisaccade Error Rate: The 2 × 3 MANOVA yielded no significant main effects of Condition (F(1, 36) = 2.855, p = .095, η2 p = .038), but Group has a significant main effects (F(2, 36) = 6.309, p = .003, η2 p = .149) on antisaccade error rate. Post-hoc comparisons revealed that participants in the motivational self-talk group had a significantly higher error rate than those in the control group (p = .005) and instructional self-talk group (p = .002). There was no significant Condition × Group interaction (F(2, 36) = 1.329, p = .271, η2 p = .036).**

**Key-Press Error Rate: The results of the 2 × 3 MANOVA on key-press error rate revealed no significant main effect of condition (F(1, 36) = 0.406, p = .526, η2 p = .006) or group (F(2, 36) = 0.216, p = .806, η2 p = .006). There was also no significant Condition × Group interaction (F(2, 36) = 0.183, p = .833, η2 p = .005).**

**Pupil Diameter: The 2 × 2 MANOVA on pupil diameter showed that no significant main effects of Condition (F(1, 36) = 1.932, p = .169, η2 p = .026), but Group has a significant main effects (F(2, 36) = 4.404, p = .016, η2 p = .109) on antisaccade pupil diameter. There was no significant Condition × Group interaction (F(2, 36) = 0.027, p = .973, η2 p = .001).**

**We also conducted a post hoc power analysis, Input:	Effect size f(V) = 0.25, α err prob = 0.05,	Total sample size = 39, Number of groups = 3, Number of measurements =	2. After calculating, Power (1-β err prob) = 0.249, this means that there is a low probability (24.9%) of correctly rejecting the null hypothesis when it is false. Therefore, MANOVA may not be an appropriate statistical method for the present research.**

p.16. I suggest the authors to report for all the analyses performed the appropriate assumptions.

**Thank you for your suggestion. We appreciate your feedback and will make sure to report the appropriate assumptions for all the statistical analyses performed in the revised manuscript. Please see more details in the section of Result of the Study 2.**

p.22. The authors are advised to include applied implications to their discussion section as this would help coaches but also sport psychologists to choose the right self-talk cues for their athletes.

Thank you for your suggestion. In the final of Discussion, I proposed ND-ST-AC **Integration Model (i.e., Noise Distraction-Self-Talk-Attentional Control Integration Model), the model integrates several factors, including noise distraction, self-talk, and attentional control, and their potential effects on cognitive load and athletic performance. The conclusion emphasizes the importance of using self-talk appropriately to balance attentional systems and maintain attentional control, particularly in the presence of noise distraction. Improper use of self-talk can disrupt the balance of attentional systems, diminish attentional control, and ultimately impact athletic performance, regardless of noise distraction.**

**References**

Abdoli, B., Hardy, J., Riyahi, J. F., & Farsi, A. (2018). A Closer Look at How Self-Talk Influences Skilled Basketball Performance. *The Sport Psychologist*, *32*(1), 9-15. https://doi.org/10.1123/tsp.2016-0162

Dali, M. S., & A. Parnabas, V. (2018). The effects of self-talk on free throw performance and the level of anxiety among male novice basketball players. *MoHE*, *7*(1). https://doi.org/10.15282/mohe.v7i1.174

Hardy, J. (2006). Speaking clearly: A critical review of the self-talk literature. *Psychology of Sport and Exercise*, *7*(1), 81-97.
