# Supplementary material for: The effect of motivational and instructional self-talk on attentional control under noise distraction
Source: PLoS One. 2023 Sep 29;18(9):e0292321. doi: 10.1371/journal.pone.0292321 (PMC10540956; doi:10.1371/journal.pone.0292321)
Supplement: Attachment — Submitted filename: Response to Reviewers 2.docx [file pone.0292321.s004.docx]

1. I seriously recommend professional language editing. I gave some examples.

L58-62. Sentence is too long and difficult to be understood.

**Thank you for your valuable feedback. I understand the sentence was initially complex and challenging to follow. I have now broken it down into several smaller sentences to clarify the meaning and improve readability. The revised sentences now read as follows: “Furthermore, anxiety escalates the allocation of attention to irrelevant stimuli, both internal and external, leading to bottom-up processing. This process affects key functions of AC (i.e., inhibition and shifting), especially inhibition, the primary indicator.” I hope this provides a clearer understanding of the concept I am presenting.**

P79. Remember that “studies” or “investigations” do not find anything, but “researchers” or “scholars” or “investigators” do!

**Thank you for your valuable feedback. I understand your point and agree that it is the researchers who make the discoveries, not the studies themselves. I have therefore revised the sentences and you could see them in revised manuscript with track changes.** **I hope this modification appropriately addresses your concern.**

L158. Please distinguish between affection and emotion. I think it should be emotion here.

**Thank you for your suggestion. In L158, I cited the article from Hardy et al. (2009). In the original text, under the heading of “Affective mechanisms,” Hardy stated: “Although there has been much debate in the sport and exercise psychology literature concerning the definition and conceptual distinction of the terms ‘affect’, ‘mood’ and ‘emotion’, we have used the phrase ‘affective mechanisms’, as an umbrella term overseeing this family of concepts.” And after further checking on APA Dictionary of Psychology, the definition of “affect” is: Any experience of feeling or emotion, ranging from suffering to elation, from the simplest to the most complex sensations of feeling, and from the most normal to the most pathological emotional reactions. Hence, I believe “affect” is the most appropriate term to use in this context, and not “emotion” or “affection”.**

Please use participants instead of subjects.

**Thank you for your suggestion. I have changed all “subjects” into “participants”, you could see them in revised manuscript with track changes.**

L202. The word "normal" you are trying to express should mean untrained, please amend it.

**Thank you for your suggestion. You are correct that the term 'normal' was not the most accurate descriptor in this context. I have now amended the sentence to specify that the participants had no prior basketball training, which is what I intended to convey with the term 'normal'. The revised sentence could be seen in revised manuscript with track changes.**

L310-319. This sentence is so long that it makes me very confused.

**Thank you for your suggestion. I have revised the sentence to improve its clarity and readability. I believe this revision provides a clearer, more understandable description of the trial process.**

2. I still think the abstract is too long, please refer to the author's guidelines for changes.

**Thank you for your suggestion. I have revised and shortened the abstract, ensuring it aligns with the guidelines provided for authors. I hope that the changes made are satisfactory and meet your requirements.**

3. Parameters should be reported with two or three decimals only.

**Thank you for your suggestion. I have kept 2 as decimals for all parameters in table two.**

4. It is also good practice to present the interaction effects first, before moving onto the main effects.

**Thank you for your suggestion. According to APA 7^th^ edition, however, reporting statistical results needs to be relevant hypotheses. In addition, presenting the results of tests should be in the order that you performed them—report the outcomes of main tests before post-hoc tests. In the present research, the first hypothesis to test is whether the noise has an effect on the AC; the second hypothesis to test is whether the self-talk has an effect on the AC. The moderate effect is tested at the end. Therefore, in order to maintain the overall logic of the article, it is necessary to keep the original order of results presentation.**

5. There are still many missing spaces, e.g., L130.

**Thank you for your suggestion.** **I have thoroughly reviewed the document and corrected this issue not only at the line you pointed out, but throughout the entire manuscript. I have taken extra care to ensure the correct use of spaces throughout the text. I appreciate your patience and attention to detail, which helps improve the clarity and readability of the manuscript.**

**References**

Hardy, J., Oliver, E., & Tod, D. (2009). A framework for the study and application of self-talk within sport. *Advances in applied sport psychology: A review*, 37-74.
