# Supplementary material for: The effect of motivational and instructional self-talk on attentional control under noise distraction
Source: PLoS One. 2023 Sep 29;18(9):e0292321. doi: 10.1371/journal.pone.0292321 (PMC10540956; doi:10.1371/journal.pone.0292321)
Supplement: Attachment — Submitted filename: Response to Reviewers 3.docx [file pone.0292321.s005.docx]

**Response to reviewers**

Dear Editor and Reviewers,

I wanted to express my heartfelt gratitude for taking the time to review my manuscript. Your insights and constructive feedback were invaluable, and I truly appreciate the expertise you brought to this review process.

Your suggestions were astute and have greatly improved the quality and clarity of the work. I have taken your feedback to heart and have made necessary revisions to address the concerns and recommendations you mentioned.

I recognize and appreciate the effort and dedication it takes to provide such thorough and insightful feedback. Please know that your input has not only improved this particular work but has also contributed to my growth as a researcher.

Thank you once again for your invaluable contribution. I look forward to possibly benefiting from your expertise in the future.

Sincerely,

Liu Yang & Yingchun Wang

Additional Editor Comments

(-1-) theory

The authors have utilised Eysenck's attention control theory of anxiety as the theoretical framework for the study. The main argument of this theory revolves around the notion that momentary (state) anxiety negatively impacts working memory functions, including executive control, phonological loop, and visual-spatial sketchpad. While this theory might be insightful and serve as a base metaphor in exploring individual differences in anxiety and related personality concepts such as neuroticism, it does not offer any specific predictions about the performance effects that could potentially be expected in the present research context. To address this limitation and enhance the theoretical impact of the study, it is recommended to provide a theoretical background that focuses on predicting the effects of the factors that are assumed to influence the outcome performance measures in the Stroop and Anti-saccade tasks. This theoretical background should address how the chosen factors, such as noise distraction and self-talk strategies, are expected to interact with attentional control and ultimately impact task performance. By incorporating a more predictive theoretical framework, the study findings and interpretations would be much better grounded and would likely contribute largely to the understanding of attentional control under noise distraction conditions. To guide you in the process of revision, I recommend a recent work of Schumann et al. which provides you with an overview of theory and relevant studies on the interplay of computational (directing, coordinating) and energetical (motivating) attentional resources to ensure performance stability (doi:10.3389/fpsyg.2022.867978), in both basic and applied (e.g., sports) psychology.

**Thank you for your suggestions regarding the theoretical foundation of this study. The reference you recommended (Schumann et al., 2022) elaborately details the impact of rest breaks on attention restoration in multitasking world. This article classifies rest, summarizes its effects on task performance, and discusses methodological issues. The explanation of phenomena through a theory is a complex and lengthy process, wherein the theory needs constant refinement. No matter it is Energetic Capacity Model, Strategic Resource Model, or other theories, most of their studies are derived from multitasking research. However, in the present study, both Experiment 1 and Experiment 2 focus on single-tasking research. The core objectives of the study are to explore the effects of noise distraction and self-talk on inhibition and their interactions, and develop Attention Control Theory (ACT). Your understanding of ACT is mostly accurate, but as mentioned, theories constantly evolve. Once a work is published, it no longer solely belongs to the author. As Roland Barthes puts it, "The death of the author is the birth of the reader." Since its inception in 2007, ACT has undergone 16 years of development (Eysenck et al., 2007). Its scope has broadened beyond just the effects of anxiety on attentional control, encompassing variables such as working memory (Angelopoulou & Drigas, 2021), mental health (Armstrong & Olatunji, 2012), affect (Pessoa, 2009). In my opinion, anxiety is one of the keys to ACT, with another key being the balance between stimulus-driven bottom-up processing and task-driven top-down processing. If the balance of attention system is disrupted, individuals might be more susceptible to external stimuli, leading to distractions and thus affecting attentional control. From the theoretical perspective, ACT can offer predictions for experimental results; from the results perspective, experimental results of the present study can further develop ACT. In conclusion, I believe ACT serves as a foundational theory for this study, but it's also essential to integrate multiple theories for interpreting the experimental results. Therefore, in the discussion section, explanations based on the Strategic Resource Model have been added (see more details in p.25).**

(-2-) design to study "self-talk" vs. design to study "cuing"

In sports psychology fields, the concept of self-talk is typically used to study real-life tasks such as tennis or basketball because in these tasks, the coordination of multiple mental operations is crucial as they need to be executed in a well-timed sequence and in the correct or optimal order. When the task is not already over-learned (or is below a certain level of automatisation), self-talk strategies are critical as they can aid coordinating action sequences. In other words, directional self-talk is equivalent to representing the points in a sequence in working memory prior to the actual execution of the action, ensuring that the intended sequence of movements is followed accurately. When the task is highly over-learned, then sole motivational self-talk is more essential (see Schumann et al., doi:10.3389/fpsyg.2022.867978, chap. 4.5) in ensuring the retrieval of specified sequences from memory (and not to miss it at some occasions). The present paper employs the concept and terminology of self-talk explicitly, while the actual experiments align more with other concepts referred to as "cueing" in the literature. The problem here is that this creates a misunderstanding as you link your experiments to the self-talk literature while the actual experiments are essentially designed as commonly done in the field of cognitive-experimental research. These studies focus on cueing effects, specifically involving directional cueing (e.g., look-away effect in the antisaccade task) and effort cueing (e.g., try-harder effect). To understand the mechanism of cueing, in particular to understand effort cuering relating to why some of the manipulations did not work in the present experimental context of your study, I would suggest one of my own work (doi:10.1007/s00426-016-0810-1), apologising for doing so, but in this work it is explained how try-harder manipulation work, what factors are critical and what aspects of performance is most indicative of increased motivation. In very brief, (1) motivational cues work best when pre-instructed (i.e., the cue try-hard is explained before the experiment to the participant), (2) when they are implemented as reminders during the task (because participants would forget the instruction over the course of a block of trials (see Schumann et al., 2022, chap. 5.4) when the instruction is only given before the experimental block), that the instruction is given in optimal temporal intervals before the task (to enable the participants to get ready (these two papers of Langner et al. and Polzien et al. are recommendable as they highlight on this aspect, the first in more general terms (doi:10.1037/xhp0000561,) and the second in the context of sports (basketball) psychology (doi:10.1037/xap0000419).

**Thank you for your valuable suggestions and recommendations. After carefully reading the literature you provided me (Langner et al., 2018; Polzien et al., 2023; Schumann et al., 2022; Steinborn et al., 2017), I have a clearer understanding of the similarities and differences between motivational self-talk and the implementation of the effort instruction. Self-talk is defined as: “(a) verbalizations or statements addressed to the self; (b) multidimensional in nature; (c) having interpretive elements association with the content of statements employed; (d) is somewhat dynamic; and (e) serving at least two functions; instructional and motivational, for the athlete (Hardy, 2006),” and effort instruction is defined as a cue to instruct participants try harder in the trial (Steinborn et al., 2017). From a definitional standpoint, hence, motivational self-talk is different from effort instruction. In addition, the two also have slight differences in some other characters, such as the context teller, frequency and performance (see Table 1). It is clear that the current paper employs 'self-talk' correctly, rather than using 'cueing' as it is typically used in the field of cognitive-experimental research. However,** **motivational self-talk and effort instruction share many similarities and have the common goal of improving motivation and performance. This is also reflected in the similarity between the result of the present study and yours (Steinborn et al., 2017).** **Specifically, try-harder instruction resulted in a global speed-up of responses, while error rate is higher than stander condition in short foreperiod (FP, the interaction effect of FP × CUE is significant), and motivational self-talk also increased the error rate in antisaccade task. Notably, the short FP and the interval between “ready” (the signal of starting self-talk) and fixation point (the signal of starting of the antisaccade task) are both 1000ms.** **Therefore, we assume that the results of present study partially confirm your conclusion from another aspect, which is effort instruction in short FP hampered the precise control of the motor-system components that are decisive for performance. Finally, thank you for your constructive suggestions and recommendations again, I will add relevant information in Discussion par**t **(see more details in p.24-25)**.

**Table 1. Characters of motivational self-talk and effort instruction**

| Characters | Motivational self-talk | Effort instruction |
| --- | --- | --- |
| Context | Cue word(s) | Cue word(s) |
| Context teller | Participants themselves | The text on screen |
| Timestamp | Beginning of a trial | Beginning of a trial |
| Frequency | Every trial | 20% of all trials |
| Interference | Not interfere any process | Not interfere any process |
| Performance | Not necessarily improve | Must improve |

(-3-) stroop task

The calculation of effects for the Stroop task in your study appears to deviate from the conventional methodological standards typically employed for Stroop-like (conflict) tasks. It is important to acknowledge that the task used in your study does not strictly adhere to the traditional Stroop test or stroop-like tests. A paper by Polzien et al., which I recommended earlier, nicely explains these aspects in the context of sports psychology (though they used a stroop-like paradigm known as the head-fake paradigm in basketball). The way performance measures are computed in your study resembles more of a reaction time effect solely for the incongruent condition, while the congruent condition is simply disregarded. In typical computerized Stroop test versions, participants are presented with colour words and are required to respond to the colour itself, with the word serving as the irrelevant dimension. There are two conditions: the congruent condition, where the word and colour are the same, and the incongruent condition, where the word and colour differ, leading to increased difficulty. The Stroop effect is usually calculated as the difference in reaction times and accuracy between these two conditions.

To ensure alignment with established methodological standards for Stroop-like tasks, it is crucial to accurately measure and interpret the Stroop effect as a key performance indicator in your study design.

**Thank you for your valuable feedback. We acknowledge that the standard Stroop effect is typically calculated by comparing reaction times and accuracy between the congruent and incongruent conditions. Regrettably, during the programming of the Stroop task, the inclusion of the congruent condition data was overlooked. I deeply regret that I cannot recalculate the data following the conventional method due to this oversight. The decision not to include the congruent condition data was motivated by several reasons: (1) Study Objective: The primary aim of Study 1 was to ascertain the effectiveness of noise distraction materials in attenuating inhibition. With this goal in mind, we believed that demonstrating a difference in accuracy or reaction time for the incongruent condition—between noisy and quiet conditions—would suffice. (2) Research Focus: While Study 2 is the main thrust of our research, Study 1 served primarily as a foundational stage for it. (3) Literature Precedent: Our method was influenced by Alimohammadi and Ebrahimi (2017). In their work, the effects of low-frequency and high-frequency noises on inhibitory performance were compared. Here, the key dependent variables were "incorrect reactions" and "working time" in the incongruent conditions of the Stroop task, and they did not calculate the Stroop effect as the conventional procedure. To address this methodological limitation, we will explicitly mention it in our manuscript. This ensures readers fully grasp the data constraints when interpreting our results. We appreciate the keen insights provided and hope to make the necessary amendments to enhance our paper's clarity and rigor (see more details in p.28).**

(-4-) statistics

the statistics including what is presented in tables is at parts incorrect or inadequate. To name one point, you use a t-test to compare the reaction times of only the incompatible trials of the stroop test with regard to the noise condition. A correct comparison would be to use the factors congruent, incongruent and noise in a three-factorial anova design separately on reaction time and errors, which would result in 7 statistical effects (3 main, 3 two-way interactions, 1 three-way interaction). To say the stroop effect is affected by noise would be validly, when there is a significant three-way interaction effect, indicating that the RT difference (the difference between congruent and incongruent trials is modulated by the factor noise). I suggest considering this in a potential revision of the manuscript.

**Thank you for your suggestion. We deeply regret and acknowledge an oversight in our experimental design. Due to this oversight, only data from the incongruent condition were included in Study 1. This means we cannot conduct ANOVA analysis as you suggested, and we deeply apologize for it. In our future studies, we will ensure rigorous experimental design checks to avoid such oversights. We believe in the importance of this line of inquiry and are committed to providing a comprehensive understanding of the topic. Thank you again for pointing out this crucial aspect. We greatly appreciate your patience and understanding.**

(-5-) providing additional information

in the method section, several aspects need more specification, to name only one among others, it would be crucial to know more about the noise manipulation with regard to duration, intensity, irrelevant speech effects, and so on. In general, I suggest presenting all information relevant to the experiments so that other researchers would potentially be able to replicate your work in a follow-up study.

**Thank you for highlighting the need for more specificity in our method section. We have expanded on the details of the noise manipulation, providing information on duration, intensity, and other relevant properties to make sure that other researchers would be able to replicate our work in the future (see more details in p.10).**

**References**

Alimohammadi, I., & Ebrahimi, H. (2017). Comparison between effects of low and high frequency noise on mental performance. *Applied Acoustics*, *126*, 131-135-135. <https://doi.org/10.1016/j.apacoust.2017.05.021>

Angelopoulou, E., & Drigas, A. (2021). Working memory, attention and their relationship: A theoretical overview. *Research, Society and Development*, *10*(5), e46410515288. <https://doi.org/10.33448/rsd-v10i5.15288>

Armstrong, T., & Olatunji, B. O. (2012). Eye tracking of attention in the affective disorders: A meta-analytic review and synthesis. *Clinical Psychology Review*, *32*(8), 704-723. <https://doi.org/10.1016/j.cpr.2012.09.004>

Eysenck, M. W., Derakshan, N., Santos, R., & Calvo, M. G. (2007). Anxiety and cognitive performance: attentional control theory. *Emotion*, *7*(2), 336. <https://doi.org/10.1080/02699931.2015.1081494>

Hardy, J. (2006). Speaking clearly: A critical review of the self-talk literature. *Psychology of Sport and Exercise*, *7*(1), 81-97.

Langner, R., Steinborn, M. B., Eickhoff, S. B., & Huestegge, L. (2018). When specific action biases meet nonspecific preparation: Event repetition modulates the variable-foreperiod effect. *Journal of Experimental Psychology: Human Perception and Performance*, *44*(9), 1313-1323. <https://doi.org/10.1037/xhp0000561>

Pessoa, L. (2009). How do emotion and motivation direct executive control? *Trends in Cognitive Sciences*, *13*(4), 160-166. <https://doi.org/10.1016/j.tics.2009.01.006>

Polzien, A., Güldenpenning, I., & Weigelt, M. (2023). Repeating head fakes in basketball: Temporal aspects affect the congruency sequence effect and the size of the head-fake effect. *Journal of Experimental Psychology: Applied*, *29*(2), 292-301. <https://doi.org/10.1037/xap0000419>

Schumann, F., Steinborn, M. B., Kürten, J., Cao, L., Händel, B. F., & Huestegge, L. (2022). Restoration of Attention by Rest in a Multitasking World: Theory, Methodology, and Empirical Evidence. *Frontiers in Psychology*, *13*. <https://doi.org/10.3389/fpsyg.2022.867978>

Steinborn, M. B., Langner, R., & Huestegge, L. (2017). Mobilizing cognition for speeded action: try-harder instructions promote motivated readiness in the constant-foreperiod paradigm. *Psychological research*, *81*(6), 1135-1151. <https://doi.org/10.1007/s00426-016-0810-1>

Reviewer #1: The authors have addressed all the comments provided by all the reviewers. I have no further comments.

Reviewer #2: The paper is interesting and almost ready for publication, but as the other reviewer commented several times it needs professional language editing. Although it needs minor revisions in many pages still has a lot of errors. Below I have spotted some and make some additional comments for the authors to improve the quality of the paper.

Abstract

These distractions disrupt the attentional systems, ultimately putting the athletes' inhibition ability and performance to the test. Please rephrase the above statement. …specifically “to the test”.

**Thank you for your suggestion. Based on your feedback, I propose the following revision: "These distractions disrupt the attentional systems, ultimately compromise the athletes' inhibition ability and directly affect their performance on the court."**

Introduction

p. 6, ln.112. Participants in the Drat-throwing task. Please correct

**Thank you for your suggestion. I apologize for the typo. This mistake has been corrected in the revised manuscript.**

p. 6, ln.117. challenge, which led to better performance on the Drat-throwing task. Please correct.

**Thank you for your suggestion. I apologize for the typo. This mistake has been corrected in the revised manuscript.**

p. 6. Lns. 108-118. I would suggest here to read and report the nice discussion about the matching hypothesis. Hatzigeorgiadis, Zourbanos, and Theodorakis (2007) noticed that depending on parameters of the task, some self-talk cues can be more effective than others, explaining the equivocal results on the matching hypothesis. I would suggest you to read Zourbanos et al. 2013 in TSP.

**Thank you for your suggestion. After reviewing the mentioned paper (Hatzigeorgiadis et al., 2007; Zourbanos et al., 2013), I found their insights further complemented my understanding, and I have referenced their work where relevant to strengthen the introduction in my manuscript (see more details in p.6, lns104-117).**

p. 6, lns. 112-120. I am not convinced about this please elaborate.

**Thank you for your suggestion.** **We have expanded on this by providing a more details, and we believe these additions provide a more comprehensive understanding of self-talk (see more details in p.7, lns 117-146).**

Results

Are the tables formatted based on PlosOne and APA recommendations? Please see the specific instructions.

**Thank you for your suggestion. The formal of tables follows “PLOS Manuscript Body Formatting Guidelines”.**

Discussion

Is there a figure inserted in the discussion? This is the first time I see a figure in the discussion section. Please delete it.

**Thank you for your question and suggestion. There indeed is a figure inserted in the discussion, aiming to visually represent the ND-ST-AC Integration Model (ND: noise distraction; ST: self-talk; AC: attentional control). We believe this figure greatly aids readers in grasping the interactions under discussion.** **Having a visual representation within the discussion can streamline the understanding of our proposed model and its implications. This approach, while perhaps less traditional, is not without precedent. For instance, Feher da Silva and Hare (2020) in their article published in Nature Human Behaviour inserted a figure in the discussion to clarify differences in the interpretation of results by two theories. Similarly, Shirer et al. (2012) also included a supplementary figure in their discussion section to elucidate anticipated improvements in imaging techniques as technology evolved. Therefore, given the figure's significance in clarifying the model's intricacies and aiding readers' understanding, we humbly request to retain it in the discussion section.**

Feher da Silva, C., & Hare, T. A. (2020). Humans primarily use model-based inference in the two-stage task. *Nature Human Behaviour*, *4*(10), 1053-1066. <https://doi.org/10.1038/s41562-020-0905-y>

Hatzigeorgiadis, A., Zourbanos, N., & Theodorakis, Y. (2007). The Moderating Effects of Self-Talk Content on Self-Talk Functions. *Journal of Applied Sport Psychology*, *19*(2), 240-251. <https://doi.org/10.1080/10413200701230621>

Shirer, W. R., Ryali, S., Rykhlevskaia, E., Menon, V., & Greicius, M. D. (2012). Decoding Subject-Driven Cognitive States with Whole-Brain Connectivity Patterns. *Cerebral Cortex*, *22*(1), 158-165. <https://doi.org/10.1093/cercor/bhr099>

Zourbanos, N., Hatzigeorgiadis, A., Bardas, D., & Theodorakis, Y. (2013). The Effects of Self-Talk on Dominant and Nondominant Arm Performance on a Handball Task in Primary Physical Education Students. *The Sport Psychologist*, *27*(2), 171-176. <https://doi.org/10.1123/tsp.27.2.171>
